# Fluctuation-driven price dynamics and investment strategies

**DOI:** 10.1371/journal.pone.0189274

**Published:** 2017-12-14

**Authors:** Yan Li, Bo Zheng, Ting-Ting Chen, Xiong-Fei Jiang

**Affiliations:** 1 Department of Physics, Zhejiang University, Hangzhou 310027, P.R. China; 2 Collaborative Innovation Center of Advanced Microstructures, Nanjing 210093, P.R. China; 3 School of Information Engineering, Ningbo Dahongying University, Ningbo 315175, P.R. China; Universidad Veracruzana, MEXICO

## Abstract

Investigation of the driven mechanism of the price dynamics in complex financial systems is important and challenging. In this paper, we propose an investment strategy to study how dynamic fluctuations drive the price movements. The strategy is successfully applied to different stock markets in the world, and the result indicates that the driving effect of the dynamic fluctuations is rather robust. We investigate how the strategy performance is influenced by the market states and optimize the strategy performance by introducing two parameters. The strategy is also compared with several typical technical trading rules. Our findings not only provide an investment strategy which extends investors’ profits, but also offer a useful method to look into the dynamic properties of complex financial systems.

## Introduction

Financial markets, as a typical complex dynamic system with many-body interactions, have drawn much attention of scientists from different fields during the past decades and much progress has been achieved [1–10]. Quantification of the price dynamics in financial markets would provide a great basis for deepening our understanding of the financial market behaviours [8, 11–20].

There have been various approaches in researches on the comprehension of financial markets. Recently, it is reported that massive data sources, such as Twitter and Google Trends, can be linked to the transaction frequency and price movements in the stock markets [21–24]. Since changes in these “big data” can be interpreted as early signals of market moves, several hypothetical strategies have been constructed for validation of this argument [25–28]. The empirical analysis of financial time series’ properties provides new insights into the non trivial nature of the stochastic process of stock prices [29–34]. Besides, some agent-based modeling methods have been proposed to investigate the role of heterogeneity of agents with respect to the price dynamics [35–41].

The temporal correlation functions can be used to characterize the dynamic properties of the financial markets [42, 43]. Since the autocorrelating time of returns is extremely short, which is on the minute time scale, our understanding on the movement of the price return itself is limited.

Understanding the driven mechanism of the price dynamics in financial markets is important and challenging. Recently, a dynamic observable nonlocal in time is constructed to explore the correlation between past volatilities and future returns [42]. This nonlocal correlation is designated as the “fluctuation-driven effect”, which may be concerning the nonstationary dynamic property of the complex systems [44]. In this paper, we construct an investment strategy to study how dynamic fluctuations drive the price movements in stock markets. We should emphasize that the fluctuation-driven effect based strategy is different from other information-driven strategies. It is constructed from the perspective of the internal price dynamics in the financial markets instead of the external information such as search volumes or investors’ sentiments. With the strategy, we not only advance our understanding to the financial markets but also provide a concrete application for financial practitioners.

According to the efficient market hypothesis [45], the strategies based on the analysis of historical price movements should not be useful because all agents were rational and able to respond promptly to all market information so that there will be no arbitrage opportunity. However, accumulating evidences are presented against this hypothesis [32, 46–49]. Some technical trading rules have been proved to be effective [50–56]. Different algorithms are utilized to forecast the price movements and quantify the price dynamics [57–60]. Various researches have suggested that trading strategies can be regarded not only as a technique to generate excessive trading profits but also as a powerful instrument to examine the traditional financial hypothesis and explore the dynamic properties of financial markets [28, 61–66].

In this paper, an investment strategy is proposed to explore the fluctuation-driven price dynamics in financial markets. The strategy provides a practical application for financial practitioners, which can be seen as an evidence to examine the efficient market hypothesis. We implement the strategy in different stock markets in the world, and study the relation between the strategy profitability and the strength of the fluctuation-driven effect. We investigate how the strategy performance is influenced by different market states and optimize the strategy performance by introducing two more parameters. The strategy is compared with several typical technical trading rules as well.

## Materials and methods

### Data retrieval

We collect the daily closing price of 20 stock market indices in the world. All the data are obtained from Yahoo! Finance (finance.yahoo.com). The time periods of the market indices are presented in Table 1. Our computation is accomplished on the platform MATLAB R2012a.

**Table 1:** The whole time period *T*, parameter *k*, annualized cumulative return of the ‘buy-and-hold’ strategy and the FDE strategy.

Index	*T*	*k*	$R_{A}^{buy-and-hold}$	$R_{A}^{FDE}$
MERV (*Argentina*)	2014.3-2016.9	0.5	0.281	0.661(60)
S&P500 (*America*)	2011.1-2015.12	0.6	0.053	0.334(25)
AXJO (*Australia*)	2012.8-2015.1	0.5	0.012	0.241(17)
BFX (*Belgium*)	2013.1-2015.7	0.5	0.127	0.362(61)
BVSP (*Brazil*)	2012.11-2015.6	0.5	0.128	0.466(34)
GSPTSE (*Canada*)	2012.6-2015.9	0.6	-0.077	0.307(18)
IPSA (*Chile*)	2012.5-2016.7	0.7	0.013	0.167(14)
SCI (*China*)	2009.4-2014.4	0.6	-0.061	0.488(33)
FTSE (*England*)	2013.1-2016.9	0.7	0.110	0.259(15)
FCHI (*France*)	2012.1-2015.9	0.6	0.032	0.506(32)
DAX (*Germany*)	2013.2-2015.7	0.5	0.153	0.412(36)
HSI (*Hongkong*)	2013.5-2015.8	0.5	0.043	0.246(19)
BSESN (*India*)	2012.10-2016.1	0.6	-0.072	0.315(19)
JKSE (*Indonesia*)	2013.11-2016.4	0.5	-0.052	0.396(22)
N225 (*Japan*)	2012.7-2016.9	0.7	-0.150	0.456(46)
KOSPI (*Korea*)	2011.6-2014.6	0.6	0.002	0.280(16)
KLSE (*Malaysia*)	2011.9-2014.5	0.5	0.072	0.165(16)
MXX (*Mexico*)	2012.8-2016.7	0.7	0.010	0.269(16)
NZ50 (*NewZealand*)	2013.10-2016.6	0.6	0.148	0.259(16)
TWII (*TaiWan*)	2013.7-2016.7	0.6	-0.069	0.241(19)

The whole time period *T*, parameter *k*, annualized cumulative return of the ‘buy-and-hold’ strategy and the FDE strategy for all the 20 stock market indices. The FDE strategy can outperform different market indices in the world.

### Volatility-return correlation nonlocal in time

The price of a financial index at time *t*′ is denoted by *p*(*t*′). The logarithmic return is defined as

$$\begin{array}{c} R\left(t^{\prime }\right)=\ln p\left(t^{\prime }\right)-\ln p\left(t^{\prime }-1\right), \end{array}$$ (1)

and the volatility is defined as *v*(*t*′) = |*R*(*t*′)|, which measures the magnitude of the price fluctuation.

To describe the volatility-return correlation nonlocal in time, a dynamic function is proposed in Ref. [42]

$$\begin{array}{c} \Delta P\left(t\right)=P^{+}\left(t\right){{|}_{\Delta v(t^{\prime })>0}-P^{+}\left(t\right)|}_{\Delta v(t^{\prime })<0}. \end{array}$$ (2)

Here the conditional probability *P*^+^(*t*)|_Δ*v*(*t*′)>0_ is the probability of *R*(*t*′ + *t*) > 0 on the condition of Δ*v*(*t*′) > 0. Correspondingly, the conditional probability *P*^+^(*t*)|_Δ*v*(*t*′)<0_ is the probability of *R*(*t*′ + *t*) > 0 for Δ*v*(*t*′) < 0. Δ*v*(*t*′) is the difference of average volatilities in two different time windows,

$$\begin{array}{c} \Delta v\left(t^{\prime }\right)=\frac{1}{T_{s}}\sum_{i=1}^{T_{s}}v\left(t^{\prime }-i+1\right)-\frac{1}{T_{l}}\sum_{i=1}^{T_{l}}v\left(t^{\prime }-i+1\right) \end{array}$$ (3)

with *T*_*l*_ ≫ *T*_*s*_. *T*_*s*_ and *T*_*l*_ are called the short window and long window, respectively.

If past volatilities and future returns do not correlate with each other, *P*^+^(*t*)|_Δ*v*(*t*′)>0_ and *P*^+^(*t*)|_Δ*v*(*t*′)<0_ should be equal, and Δ*P*(*t*) should be zero. If Δ*P*(*t*) is computed to be non-zero, there should exist a non-zero volatility-return correlation and such a correlation is nonlocal in time.

The time windows *T*_*s*_ and *T*_*l*_ are crucial in the calculation of Δ*P*(*t*). *T*_*s*_ represents the period of time with which investors measure the fluctuation of current prices. *T*_*l*_ reflects the auto-correlating time of the dynamic fluctuations in stock markets, which is used to estimate the background volatilities. *T*_*s*_ should be much smaller than *T*_*l*_. In our calculations, *T*_*s*_ ranges from 1 to 44 days. *T*_*l*_ ranges from 45 to 250 days. Each pair of *T*_*s*_ and *T*_*l*_ is called a time window pair.

#### Computation of the conditional probabilities

We present the computation of *P*^+^(*t*)|_Δ*v*(*t*′)>0_ and *P*^+^(*t*)|_Δ*v*(*t*′)<0_ in this section. The length of the time series to calculate Δ*P* is denoted by *T*′. According to the definition of Δ*v*(*t*′), *T*_*l*_ stands for the past *T*_*l*_ days before time *t*′, which is the long time window; and *T*_*s*_ stands for the past *T*_*s*_ days before time *t*′, which is the short time window. With *t*′ ranging from *T*_*l*_ to *T*′ − *t*, we count the number of *t*′ of the following case:

- Δ*v*(*t*′) > 0
- Δ*v*(*t*′) > 0 and *R*(*t*′ + *t*) > 0
- Δ*v*(*t*′) < 0
- Δ*v*(*t*′) < 0 and *R*(*t*′ + *t*) > 0

and denote them by *N*_+_, $N_{+}^{+}$, *N*_−_ and $N_{-}^{+}$ respectively.

Then the probability of *R*(*t*′ + *t*) > 0 on the condition of Δ*v*(*t*′) > 0 is

$$\begin{array}{c} P^{+}{\left(t\right)|}_{\Delta v(t^{\prime })>0}=N_{+}^{+}/N_{+} \end{array}$$ (4)

and the probability of *R*(*t*′ + *t*) > 0 on the condition of Δ*v*(*t*′) < 0 is

$$\begin{array}{c} P^{+}{\left(t\right)|}_{\Delta v(t^{\prime })<0}=N_{-}^{+}/N_{-} \end{array}$$ (5)

The typical behavior of Δ*P* is provided in Fig 1 of Ref. [42]. In this paper, as a first approach, we take *t* = 1 and denote *AP* = Δ*P*(1), to construct our strategy.

### Construction of the strategy

As returns represent the price changes, and volatilities measure the fluctuations of the price movement, the volatility-return correlation nonlocal in time can be regarded as a description of how the price movements are driven by the nonlocal fluctuations. The nonlocal correlation is thus designated as the “fluctuation-driven effect (FDE)” to the price dynamics. In this paper, we construct a FDE strategy to further investigate the driven mechanism of the price dynamics in financial markets.

In the construction of the FDE strategy, there are several time variables and parameters. *T*_*s*_ and *T*_*l*_ are the short time and long time windows respectively for computing Δ*v*(*t*′); *t*′ in Δ*v*(*t*′) stands for time *t*′; *t* in Δ*P*(*t*) is the time lag of the correlation between *v*(*t*′) and *r*(*t*′ + *t*).

According to the previous results, Δ*P* is positive for most stock market indices in the world [42]. The positive Δ*P* is practically corresponding to that the volatilities in the past period of time enhance the positive returns in future times. Thus the FDE strategy can be expressed as follows: at time *t*′, if Δ*v*(*t*′) > 0, a buy signal will be generated. Correspondingly, if Δ*v*(*t*′) < 0, a sell signal will be generated.

To demonstrate the feasibility of this strategy, we divide the whole data series into two parts: training period and testing period. The former is used to determine the parameters of our strategy and the latter to test the strategy performance. The length of the whole data series, the training period, the testing period are denoted by *T*, *T*′, and *L* respectively. We set a parameter *k* to quantify the ratio of the length of the training period to the whole data series, i.e., *k* = *T*′/*T*. The range of *k* is from 0.5 to 0.7 in our computations, and *T* is about 3 to 5 years. Both *T* and *k* are shown in Table 1. We compute *AP* from the training period with each time window pair of *T*_*s*_ and *T*_*l*_, and then fix the time window pair of our strategy by the maximum |*AP*|.

Then the strategy is implemented on the testing period. At day *t*′, Δ*v*(*t*′) is calculated with the fixed *T*_*s*_ and *T*_*l*_. The cumulative return of the strategy is denoted by *R*_*c*_(*t*′) [27]. The investor’s behaviour is as follows:

- If Δ*v*(*t*′) > 0, the investor buys the market index at the closing price *p*(*t*′) on day *t*′ and sells it at price *p*(*t*′ + 1). In this case, *R*_*c*_(*t*′ + 1) = *R*_*c*_(*t*′) + *R*(*t*′ + 1).
- If Δ*v*(*t*′) < 0, the investor sells the market index at the closing price *p*(*t*′) on day *t*′ and buys it back at price *p*(*t*′ + 1). In this case, *R*_*c*_(*t*′ + 1) = *R*_*c*_(*t*′) − *R*(*t*′ + 1).

Initially, *R*_*c*_ is set to be zero. *R*_*c*_ at *t*′ represents the increase of the value from the investor’s initial assets with the FDE strategy. When the investor decides to buy or sell, all his money is used up to buy or all his assets are sold out. It should be noted that if *AP* of the training period is negative, we ought to reverse our strategy: if Δ*v*(*t*′) > 0, a sell signal will be generated; if Δ*v*(*t*′) < 0, a buy signal will be generated.

## Results

### Feasibility of the FDE strategy

**Fig 1:**
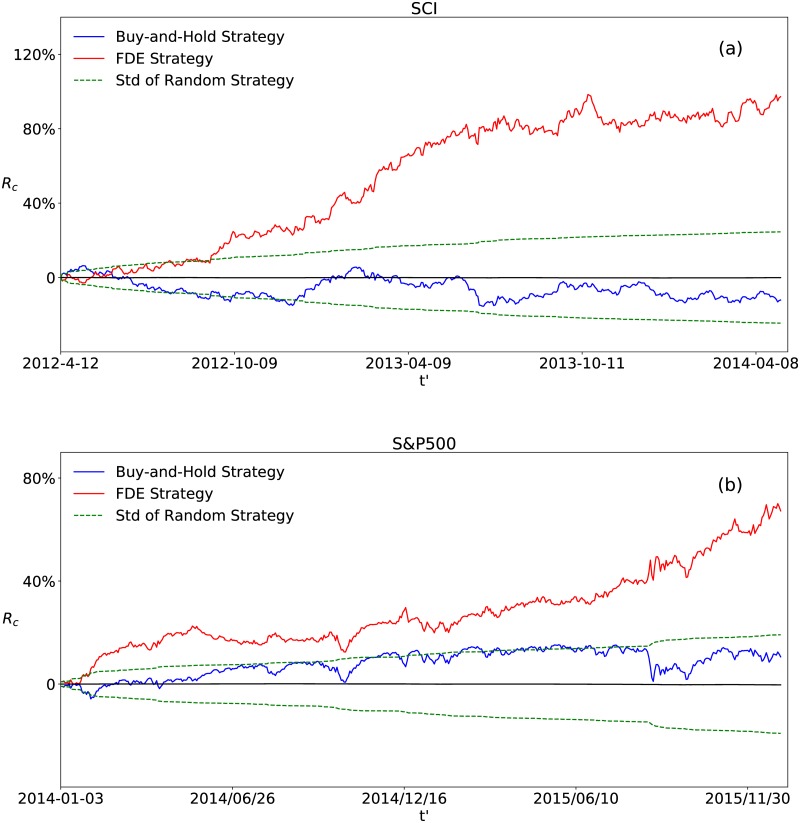
Performance of the FDE strategy. Cumulative return for (a) the SCI and (b) the S&P500. *R*_*c*_ of the FDE strategy is plotted in red line. It is compared to the ‘buy-and-hold’ strategy plotted in blue line and the standard deviation of 10,000 simulations with a random strategy displayed in dashed green lines. Here $\langle R_{c}^{random}\rangle =0$.

We implement the FDE strategy on different stock market indices in the world. The result of the Shanghai Composite Index (SCI) and the Standard&Poor’s 500 Index (S&P500) is shown in Fig 1. We compare the performance of the FDE strategy with two benchmark strategies, the random strategy and the ‘buy-and-hold’ strategy.

In Fig 1, the ‘buy-and-hold’ strategy stands for buying the market index at the beginning of the trading time period, holding, and then selling it at the end. Thus, the cumulative return *R*_*c*_ of the ‘buy-and-hold’ strategy is simply computed by *R*_*c*_(*t*′ + 1) = *R*_*c*_(*t*′) + *R*(*t*′ + 1). In the random strategy, the probability that the index will be bought or sold is always 50%, and the trading decision is unaffected by decisions in previous days. Correspondingly, *R*_*c*_ of the random strategy is computed by *R*_*c*_(*t*′ + 1) = *R*_*c*_(*t*′) + *R*(*t*′ + 1) when it buys, and *R*_*c*_(*t*′ + 1) = *R*_*c*_(*t*′) − *R*(*t*′ + 1) when it sells. We report the standard deviation of the cumulative returns derived from 10,000 independent simulations of the random strategy. The mean cumulative return of the 10,000 uncorrelated random strategies is zero.

In Fig 1(a), the cumulative return *R*_*c*_ of the FDE strategy for the SCI is 97.2% for two years, with *k* = 0.6, *T*_*s*_ = 1, *T*_*l*_ = 50. Compared to -12.1% of the ‘buy-and-hold’ strategy, the strategy yields considerable profit. We perform the same computation for the S&P500. As displayed in Fig 1(b), the ultimate *R*_*c*_ is 67.3% for two years with *k* = 0.6, *T*_*s*_ = 25, *T*_*l*_ = 245. It is not as much as the SCI, but still promising compared to 10.6% of the ‘buy-and-hold’ strategy. The reason may be that the American stock market is highly developed, with large market size and complicated derivative financial tools, while the Chinese stock market is emerging and of small market size, in which the derivative financial tools are relatively basic and simple. The American stock market is more efficient so that investors are not able to make profits easily.

As shown in Table 1, the strategy is also implemented in other 18 stock markets. We adopt the annualized cumulative return *R*_*A*_ in order to compare the results in different stock markets, which is defined as

$$\begin{array}{c} R_{A}=R_{c}\times 250/L, \end{array}$$ (6)

where *L* is the length of the testing time period, *L* = *T* × (1 − *k*), and 250 represents the number of trading days in a year.

The results demonstrate that the FDE strategy can outperform different market indices in the world, which indicate that the fluctuation-driven effect is rather robust.

In order to investigate the relation between the fluctuation-driven effect and the strategy performance, we compute *AP* and *R*_*c*_ with different time windows pairs *T*_*s*_ and *T*_*l*_. Here *T*_*s*_ ranges from 1 to 44, *T*_*l*_ ranges from 45 to 250.

**Fig 2:**
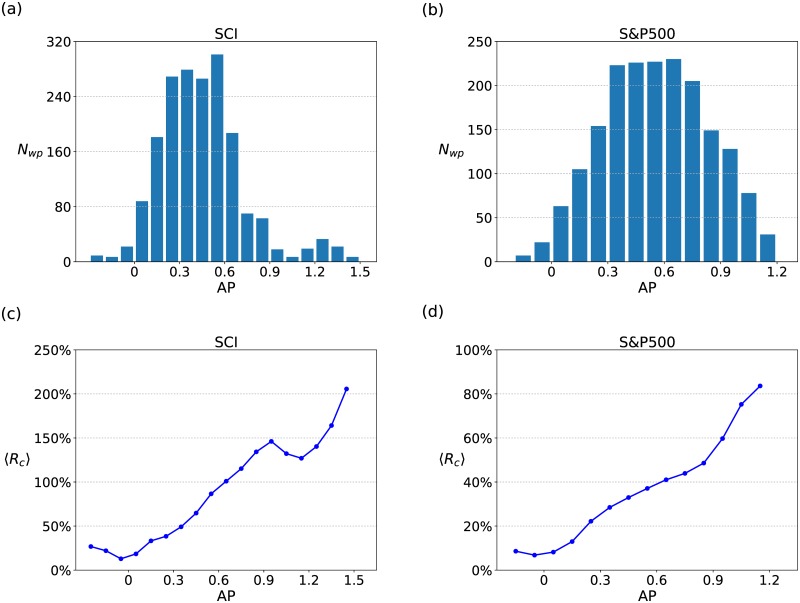
Mean cumulative returns corresponding to different AP. The distributions of the amounts of time window pairs corresponding to different *AP* for (a)the SCI and (b)the S&P500. The mean cumulative return corresponding to different *AP* for (c)the SCI and (d)the S&P500.

The distributions of the window pairs corresponding to different *AP* are shown in Fig 2(a) and 2(b) for the SCI and the S&P500 respectively. As displayed in the figure, only a few time window pairs correspond to the large *AP*. These window pairs can be regarded as the key quantities to characterize the fluctuation-driven dynamic properties of the financial markets.

The mean cumulative returns of the FDE strategy corresponding to different *AP* are shown in Fig 2(c) and 2(d). In Fig 2(c) and 2(d), 〈*R*_*c*_〉 generally increases as *AP* increases for both the SCI and the S&P500. Compared to the American stock market, the FDE strategy performs better in the Chinese stock market. These results provide us an intuitive understanding of the fluctuation-driven effect.

### Strategy performance in different market states

To address the question whether the strategy performs asymmetrically in the volatile and the stable market states, we separate the strategy into two parts. In one part we trade only if Δ*v* > 0 and in the other part only if Δ*v* < 0, corresponding to the volatile and stable market state respectively. Then we compute the winning percentage *A*_*s*_ for these two parts with different time windows pairs *T*_*s*_ and *T*_*l*_. The winning percentage of the strategy is defined as

$$\begin{array}{c} A_{s}=\frac{N^{+}{|}_{R>0}}{N}, \end{array}$$ (7)

where *N*^+^ refers to the number of transactions that bring positive strategy returns. We only consider the time window pairs which satisfy the condition *AP* > 1.2〈|*AP*|〉 for the SCI, and *AP* > 1.5〈|*AP*|〉 for the S&P500.

**Fig 3:**
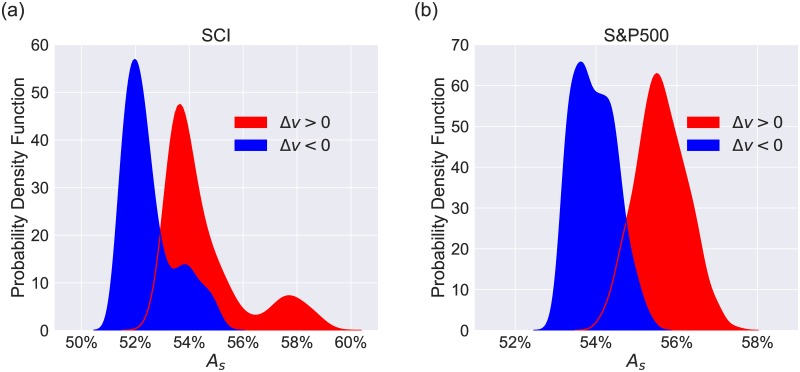
Probability density functions of the winning percentage for the volatile and stable market states. (a) The distributions of *A*_*s*_ for the two parts of the FDE strategy, one for Δ*v* > 0(red shade) and the other for Δ*v* < 0(blue shade) for the SCI. (b) A parallel analysis for the S&P500.

In Fig 3, we show the probability density functions of *A*_*s*_ for the two parts of the FDE strategy. 〈*A*_*s*_|_Δ*v*>0_〉 = 54.5% and 〈*A*_*s*_|_Δ*v*<0_〉 = 52.5% for the SCI which can be seen in Fig 3(a). The difference indicates the strategy performs better when Δ*v* > 0 rather than Δ*v* < 0. A similar result is obtained for the S&P500 in Fig 3(b), with 〈*A*_*s*_|_Δ*v*>0_〉 = 55.6% and 〈*A*_*s*_|_Δ*v*<0_〉 = 54.0%. Our result suggests that the fluctuation-driven effect is not symmetric in different market states, but it is stronger when the market state is more volatile.

Parallel computations are performed for other 18 stock market indices. The strategy is more profitable when Δ*v* > 0 in most stock markets, which consolidates our result that the volatile market state conduces more to the fluctuation-driven effect. It should be noted that despite the existence of the asymmetry of this effect, *A*_*s*_|_Δ*v*>0_ and *A*_*s*_|_Δ*v*<0_ are both more than 50% in all the markets, which indicates that our strategy is quite robust.

### Optimization of the strategy

In the previous results, Δ*P* is weak for some stock markets or some certain periods of time. To enhance it and quantify to which degree the strategy performance can be affected by the market state, we introduce two more parameters *α*_+_ and *α*_−_ to characterize the trading signal Δ*v*.

We previously constructed Δ*v* by comparing the average volatility over *T*_*s*_ with an average volatility over a longer period of time *T*_*l*_. Now we introduce Δ*v*_*α*_ to quantify how volatile the market is,

$$\begin{array}{c} \Delta v_{\alpha }\left(t^{\prime }\right)={\left\langle v\left(t^{\prime }\right)\right\rangle }_{T_{s}}-\alpha \cdot {\left\langle v\left(t^{\prime }\right)\right\rangle }_{T_{l}}. \end{array}$$ (8)

For Δ*v*_*α*_(*t*′) > 0, the larger *α* is, the more volatile the market in *T*_*s*_ is; for Δ*v*_*α*_(*t*′) < 0, the smaller *α* is, the more stable the market in *T*_*s*_ is.

Accordingly, we can update the construction of the strategy in the following way:

- If Δ*v*_*α*_+__(*t*′) > 0, we buy the market index at the closing price *p*(*t*′) on day *t*′ and sell it at price *p*(*t*′ + 1).
- If Δ*v*_*α*_−__(*t*′) < 0, we sell the market index at the closing price *p*(*t*′) on day *t*′ and buy it back at price *p*(*t*′ + 1).
- If Δ*v*_*α*_+__(*t*′) < 0 and Δ*v*_*α*−_(*t*′) > 0, we neither buy nor sell.

Considering that the price in financial markets generally fluctuates within a certain range, we let *α*_+_ range from 1 to 1.5, and *α*_−_ range from 0.5 to 1, which covers most situations.

For each pair of *α*_+_ and *α*_−_, we compute the strategy winning percentage *A*_*s*_ with different time window pairs *T*_*s*_ and *T*_*l*_. We take an average of *A*_*s*_ for the window pairs which satisfy the condition *AP* > 1.2〈|*AP*|〉 for the SCI, and *AP* > 1.5〈|*AP*|〉 for the S&P500.

**Fig 4:**
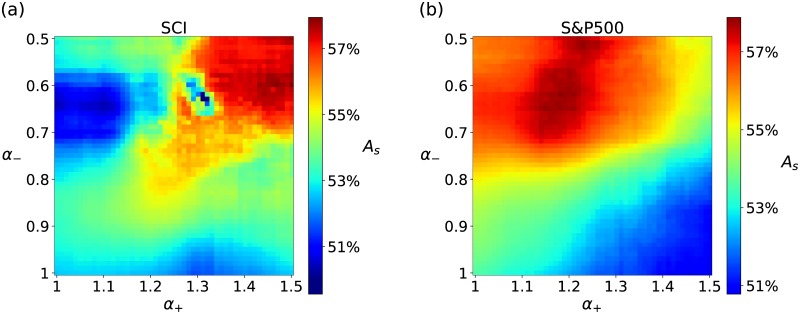
The winning percentage with different *α*_+_ and *α*_−_. *A*_*s*_ computed with different *α*_+_ and *α*_−_ for (a) the SCI and (b) the S&P500. *α*_+_ ranges from 1 to 1.5 and *α*_−_ ranges from 0.5 to 1.

It is shown in Fig 4(a) that for the SCI, *A*_*s*_ is promoted as *α*_+_ increases and *α*_−_ decreases. The highest *A*_*s*_ arises at *α*_+_ = 1.5 and *α*_−_ = 0.6. As for the S&P500 in Fig 4(b), the optimum choice is *α*_+_ = 1.18 and *α*_−_ = 0.58. The new parameters are obviously effective in improving the strategy profitability for both two market indices. It should be pointed out that the trading frequency would be reduced by introducing *α*_+_ and *α*_−_. However, the FDE strategy can still be regarded as a part of a hybrid strategy, combining with other trading rules to improve the overall performance in financial markets.

### Comparison with typical trading rules

The FDE strategy can be regarded as a concrete application of the nonlocal volatility-return correlation. It is crucial to examine its performance and compare it with other trading strategies. Here we adopt three widely used technical trading rules [52]. The trading indicators are Relative Strength Index (RSI), Moving Average Convergence Divergence (MACD) and Momentum.

#### Algorithm of the technical trading rules

- RSI: The RSI is an indicator that shows the strength of the asset price by comparison of the individual upward or downward movements of the consecutive prices. Its value is determined as
  $$\begin{array}{c} RSI_{n}\left(t^{\prime }\right)=\frac{\sum_{j=0}^{n-1}{\left[p\left(t^{\prime }-j\right)-p\left(t^{\prime }-j-1\right)\right]}_{p\left(t^{\prime }-j\right)>p\left(t^{\prime }-j-1\right)}}{\sum_{j=0}^{n-1}\left|p\left(t^{\prime }-j\right)-p\left(t^{\prime }-j-1\right)\right|}\times 100, \end{array}$$ (9)
  where *RSI*_*n*_(*t*′) is the relative strength index at time *t*′, *p*(*t*′) is the price of index at time *t*′ and *n* is the number of RSI periods. In this paper, the 14-day RSI is studied, which is a popular length utilized by traders. When *RSI*(*t*′) > 30 ≥ *RSI*(*t*′ − 1), a buy signal is generated; when *RSI*(*t*′) > 70 ≥ *RSI*(*t*′ − 1), a sell signal is generated.
- MACD: The MACD is designed mainly to identify the trend changes of the asset price. It is calculated by subtracting a longer Exponential Moving Average (EMA) from a shorter EMA, which is defined as
  $$\begin{array}{c} MACD\left(t^{\prime }\right)=EMA_{ds}\left(t^{\prime }\right)-EMA_{dl}\left(t^{\prime }\right), \end{array}$$ (10)
  where *ds* = 12 and *dl* = 26, which are the most commonly used short and long-period EMAs.
  In addition, we use a sign in order to generate the buy and sell signal of MACD. It is defined as
  $$\begin{array}{c} S_{MACD}\left(t^{\prime }\right)=\frac{1}{n}\sum_{j=0}^{n-1}MACD\left(t^{\prime }-j\right), \end{array}$$ (11)
  where *n* = 9. In our study, when *MACD*(*t*′) < *S*_*MACD*_(*t*′) < 0, a buy signal is generated; when *MACD*(*t*′) > *S*_*MACD*_(*t*′) > 0, a sell signal is generated.
- Momentum: The Momentum is an indicator that measures the strength of the tendency of an index or a stock, and it expresses the variation of the price in a concrete period of time.
  The Momentum is represented by a difference, which is defined as
  $$\begin{array}{c} M_{n}\left(t^{\prime }\right)=p\left(t^{\prime }\right)-p\left(t^{\prime }-n+1\right), \end{array}$$ (12)
  where *p*(*t*′) is the price of the index at time *t*′. As standard, we take *n* = 12 in this paper. A buy signal is generated if *M*(*t*′) > 0 ≥ *M*(*t*′ − 1). A sell signal is generated if *M*(*t*′) < 0 ≤ *M*(*t*′ − 1).

For all the trading rules, their cumulative return is computed in the following way: *R*_*c*_(*t*′ + 1) = *R*_*c*_(*t*′) + *R*(*t*′ + 1) when they buy, and *R*_*c*_(*t*′ + 1) = *R*_*c*_(*t*′) − *R*(*t*′ + 1) when they sell.

**Fig 5:**
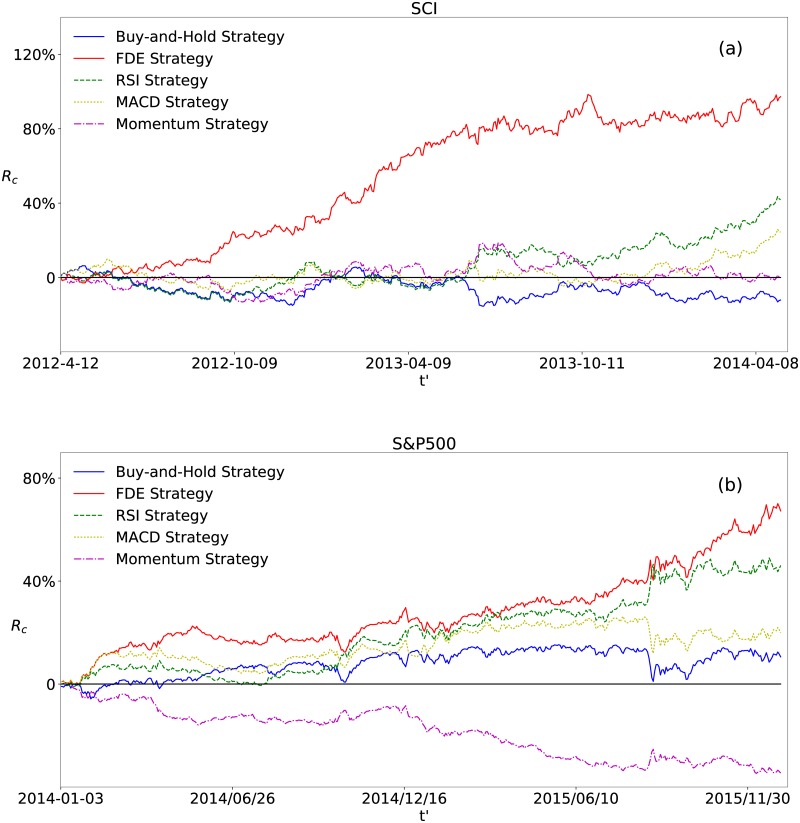
Comparison of the FDE strategy and other strategies. Cumulative return of the FDE strategy and other three technical trading rules for (a) the SCI and (b) the S&P500.

The comparison of different strategy performances is displayed in Fig 5. The FDE strategy outperforms these three trading rules for both the SCI and the S&P500. In Ref. [62], the comparison of the technical strategies and the random strategy is provided. It is shown that the profitabilities of the technical strategies and the random strategy are both around 50%, consistent with our computations. The better performance of the FDE strategy proves the effectiveness and reliability of our strategy.

If a trading rule can generate excess returns over the simple buy-and-hold policy, it serves as an evidence against the efficient market hypothesis. All these three technical trading rules, however, originate from the practical experience of the financial investors. There does not seem to be a clear dynamic mechanism related to the construction of these trading rules. In contrast, the FDE strategy is based on the volatility-return correlation nonlocal in time, which may be concerning the nonstationary dynamic property of the complex systems. This correlation is a robust and intrinsic property widely observed in different complex dynamic systems. In this sense, the FDE strategy provides us a new perspective into the understanding of the complex financial systems.

## Conclusion

In summary, we construct an investment strategy to explore how dynamic fluctuations drive the price movements in complex financial systems. The strategy is based on the volatility-return correlation nonlocal in time, which is designated as the “fluctuation-driven effect” to the price dynamics. The strategy provides a concrete application for the financial investors, which is effective in most stock markets. The profitability of the strategy can be enhanced by the strength of the fluctuation-driven effect. It is illustrated that the volatile market state leads to a better performance of the strategy. Further, We introduce two parameters *α*_+_ and *α*_−_ to describe the fluctuation-driven effect, and the winning percentage of the strategy *A*_*s*_ is promoted with large *α*_+_ and small *α*_−_. In addition, it is shown that the fluctuation-driven effect based strategy can outperform several typical technical trading rules.

These findings provide us a new insight into the fluctuation-driven price dynamics of stock markets. The volatility-return correlation nonlocal in time is a robust and intrinsic property concerning the control of the price movements, which is widely observed in different complex dynamic systems [44]. Through constructing a strategy, we investigate the properties of the fluctuation-driven effect and offer a practical significance to it. Besides, various nonlocal correlation functions in other complex dynamic systems are to be explored.

## Supporting information

S1 File Participant data. All relevant data are within the Supporting Information files.
(RAR)

## References

[1] Mantegna RN, Stanley HE. Scaling behaviour in the dynamics of an ecnomic index. Nature. 1995;376:46–49. doi: 10.1038/376046a0

[2] Lux T, Marchesi M. Scaling and criticality in a stochastic multi-agent model of a financial market. Nature. 1999;397:498–500. doi: 10.1038/17290

[3] Gabaix X, Gopikrishnan P, Plerou V, Stanley HE. A theory of power-law distributions in financial market fluctuations. Nature. 2003;423:267–270. doi: 10.1038/nature01624 12748636 10.1038/nature01624

[4] Kiyono K, Struzik ZR, Yamamoto Y. Criticality and Phase Transition in Stock-Price Fluctuations. Phys Rev Lett. 2006;96:1–4. doi: 10.1103/PhysRevLett.96.068701 10.1103/PhysRevLett.96.068701 16606055

[5] Krawiecki A, Holyst JA, Helbing D. Volatility Clustering and Scaling for Financial Time Series due to Attractor Bubbling. Phys Rev Lett. 2002;89:158701 doi: 10.1103/PhysRevLett.89.158701 12366029 10.1103/PhysRevLett.89.158701

[6] Bouchaud JP, Matacz A, Potters M. Leverage effect in financial markets: The retarded volatility model. Phys Rev Lett. 2001;87:228701 doi: 10.1103/PhysRevLett.87.228701 11736431 10.1103/PhysRevLett.87.228701

[7] Plerou V, Gopikrishnan P, Rosenow B, Amaral LAN, Stanley HE. Universal and Nonuniversal Properties of Cross Correlations in Financial Time Series. Phys Rev Lett. 1999;83:1471–1474. doi: 10.1103/PhysRevLett.83.1471

[8] Botta F, Moat HS, Stanley HE, Preis T. Quantifying Stock Return Distributions in Financial Markets. PLoS One. 2015;10:e0135600 doi: 10.1371/journal.pone.0135600 26327593 10.1371/journal.pone.0135600 PMC4556674

[9] Petersen AM, Wang F, Havlin S, Stanley HE. Market dynamics immediately before and after financial shocks: quantifying the Omori, productivity, and Bath laws. Phys Rev E. 2010;82:036114. 10.1103/PhysRevE.82.036114 21230146

[10] Chakraborti A, Toke IM, Patriarca M, Abergel F. Econophysics Review: I. Empirical facts. Quant Financ. 2011;11:991–1012. doi: 10.1080/14697688.2010.539248

[11] Zheng B, Jiang XF, Ni PY. A mini-review on econophysics: Comparative study of Chinese and western financial markets. Chin Phys B. 2014;23:078903 doi: 10.1088/1674-1056/23/7/078903

[12] Jiang XF, Zheng B, Qiu T, Ren F. Extreme-volatility dynamics in crude oil markets. Eur Phys J B. 2017;90:30 doi: 10.1140/epjb/e2017-70482-4

[13] Liu L, Wei JR, Huang JP. Scaling and Volatility of Breakouts and Breakdowns in Stock Price Dynamics. PLoS One. 2013;8:e82771 doi: 10.1371/journal.pone.0082771 24376577 10.1371/journal.pone.0082771 PMC3871165

[14] Voit J. The statistical mechanics of financial markets. Phys Today. 2005;55:51–52.

[15] Preis T. Simulating the Microstructure of Financial Markets. J Phys Conf Ser. 2010;221:012019 doi: 10.1088/1742-6596/221/1/012019

[16] Friedrich R, Peinke J, Renner C. How to Quantify Deterministic and Random Influences on the Statistics of the Foreign Exchange Market. Phys Rev Lett. 2000;84:5224 doi: 10.1103/PhysRevLett.84.5224 10990908 10.1103/PhysRevLett.84.5224

[17] Shen J, Zheng B. Cross-correlation in financial dynamics. Europhys Lett. 2009;86:48005 doi: 10.1209/0295-5075/86/48005

[18] Qiu T, Zheng B, Chen G. Financial networks with static and dynamic thresholds. Europhys Lett. 2010;12:043057.

[19] Jiang XF, Zheng B. Anti-correlation and subsector structure in financial systems. Europhys Lett. 2012;97:48006 doi: 10.1209/0295-5075/97/48006

[20] Jiang XF, Chen TT, Zheng B. Structure of local interactions in complex financial dynamics. Sci Rep. 2014;4:5321 doi: 10.1038/srep05321 24936906 10.1038/srep05321 PMC4060508

[21] Bollen J, Mao H, Zeng XJ. Twitter mood predicts the stock market. J Comput Sci. 2011;2:1–8. doi: 10.1016/j.jocs.2010.12.007

[22] Choi H, Varian H. Predicting the Present with Google Trends. Econ Rec. 2011;88:2–9. doi: 10.1111/j.1475-4932.2012.00809.x

[23] Alanyali M, Moat HS, Preis T. Quantifying the Relationship Between Financial News and the Stock Market. Sci Rep. 2013;3:3578 doi: 10.1038/srep03578 24356666 10.1038/srep03578 PMC3868958

[24] Piskorec M, Antov-Fantulin N, Novak PK, Mozetic I, Grcar M, Vodenska I, Smuc T. Cohesiveness in Financial News and its Relation to Market Volatility. Sci Rep. 2014;4:5038 doi: 10.1038/srep05038 24849598 10.1038/srep05038 PMC4030282

[25] Kristoufek L. Can Google Trends search queries contribute to risk diversification. Sci Rep. 2013;3:2713 doi: 10.1038/srep02713 24048448 10.1038/srep02713 PMC3776958

[26] Preis T, Moat HS, Stanley HE. Quantifying Trading Behavior in Financial Markets Using Google Trends. Sci Rep. 2013;3:1684 doi: 10.1038/srep01684 23619126 10.1038/srep01684 PMC3635219

[27] Moat HS, Curme C, Avakian A, Kenett DY, Stanley HE, Preis T. Quantifying Wikipedia Usage Patterns Before Stock Market Moves. Sci Rep. 2013;3:1801 doi: 10.1038/srep01801

[28] Heiberger RH. Collective Attention and Stock Prices: Evidence from Google Trends Data on Standard and Poor’s 100. PLoS One. 2015;10:e0135311 doi: 10.1371/journal.pone.0135311 26258498 10.1371/journal.pone.0135311 PMC4530949

[29] Viens F. Stochastic processes: from physics to finance. J Am Stat Assoc. 2002;97:1209–1210. doi: 10.1198/jasa.2002.s241

[30] Laloux L, Cizeau P, Potters M, Bouchaud JP. Noise Dressing of Functional Correlation Matrices. Phys Rev Lett. 1999;83:1467 doi: 10.1103/PhysRevLett.83.1467

[31] Plerou V, Gopikrishnan P, Rosenow B, Amaral LAN, Guhr T, Stanley HE. Random matrix approach to cross correlations in financial data. Phys Rev E. 2002;65:1–18. doi: 10.1103/PhysRevE.65.066126 10.1103/PhysRevE.65.066126 12188802

[32] Brock W, Lakonishok J, LeBaron B. Simple Technical Trading Rules and the Stochastic Properties of Stock Returns. J Financ. 2000;47:1731–1767. doi: 10.1111/j.1540-6261.1992.tb04681.x

[33] Podobnika B, Grossea I, Stanley HE. Stochastic processes with power-law stability and a crossover in power-law correlations. Physica A. 2002;316:153–159. doi: 10.1016/S0378-4371(02)01023-3

[34] Podobnika B, Ivanov PC, Jazbinsek V, Trontelj Z, Stanley HE, Grosse I. Power-law correlated processes with asymmetric distributions. Phys Rev E. 2005;316:153–159. 10.1103/PhysRevE.71.025104 15783366

[35] Chen JJ, Zheng B, Tan L. Agent-Based Model with Asymmetric Trading and Herding for Complex Financial Systems. PLoS One. 2013;8:e79531 doi: 10.1371/journal.pone.0079531 24278146 10.1371/journal.pone.0079531 PMC3835857

[36] Chen TT, Zheng B, Li Y, Jiang XF. New approaches in agent-based modeling of complex financial systems. Front Phys. 2017;12:128905 doi: 10.1007/s11467-017-0661-2

[37] Farmer JD, Foley D. The economy needs agent-based modeling. Nature. 2009;460:685–686. doi: 10.1038/460685a 19661896 10.1038/460685a

[38] Bertella MA, Pires FR, Feng L, Stanley HE. Confidence and the Stock Market: An Agent-Based Approach. PLoS One. 2014;9:e83488 doi: 10.1371/journal.pone.0083488 24421888 10.1371/journal.pone.0083488 PMC3885419

[39] Gontis V, Kononovicius A. Consentaneous Agent-Based and Stochastic Model of the Financial Markets. PLoS One. 2014;9:e102201 doi: 10.1371/journal.pone.0102201 25029364 10.1371/journal.pone.0102201 PMC4100891

[40] Bouchaud JP, Mezard M, Potters M. Statistical Properties of Stock Order Books: Empirical Results and Models. Quant Financ. 2002;2:251–256. doi: 10.1088/1469-7688/2/4/301

[41] Samanidou E, Zschischang E, Stauffer D, Lux T. Agent-based models of financial markets. Rep Prog Phys. 2007;70:409 doi: 10.1088/0034-4885/70/3/R03

[42] Tan L, Zheng B, Chen JJ, Jiang XF. How volatilities nonlocal in time affect the price dynamics in complex financial systems. PLoS One. 2015;10:e0118399 doi: 10.1371/journal.pone.0118399 25723154 10.1371/journal.pone.0118399 PMC4344208

[43] Qiu T, Zheng B, Ren F, Trimper S. Return-volatility correlation in financial dynamics. Phys Rev E. 2006;73:065103 doi: 10.1103/PhysRevE.73.065103 10.1103/PhysRevE.73.065103 16906892

[44] Chen TT, Zheng B, Tan L, Li Y, Jiang XF. Non-stationary effects in complex systems. Submitted for publication. 2017.

[45] Malkiel BG, Fama EF. Efficient Capital Markets: A Review of Theory and Empirical Work. J Financ. 1970;25:383–417. doi: 10.2307/2325486

[46] Ratner M, Leal R. Tests of technical trading strategies in the emerging equity markets of Latin America and Asia. J Bank Financ. 1999;23:1887–1905. doi: 10.1016/S0378-4266(99)00042-4

[47] Kwon KY, Kish RJ. Technical trading strategies and return predictability: NYSE. Appl Financ Econ. 2002;12:639–653. doi: 10.1080/09603100010016139

[48] Menkhoff L. The use of technical analysis by fund managers: International evidence. J Bank Financ. 2010;34:2573–2586. doi: 10.1016/j.jbankfin.2010.04.014

[49] Osler CL. Currency Orders and Exchange Rate Dynamics: An Explanation for the Predictive Success of Technical Analysis. J Financ. 2003;58:1791–1820. doi: 10.1111/1540-6261.00588

[50] Shi HL, Jiang ZQ, Zhou WX. Profitability of Contrarian Strategies in the Chinese Stock Market. PLoS One. 2015;10:e0137892 doi: 10.1371/journal.pone.0137892 26368537 10.1371/journal.pone.0137892 PMC4569377

[51] Chong TTL, Ng WK. Technical analysis and the London stock exchange: testing the MACD and RSI rules using the FT30. Appl Econ Lett. 2008;15:1111–1114. doi: 10.1080/13504850600993598

[52] Rosillo R, Fuente D, Brugos JAL. Technical analysis and the Spanish stock exchange: testing the RSI, MACD, momentum and stochastic rules using Spanish market companies. Appl Econ. 2012;45:12:1541–1550. doi: 10.1080/00036846.2011.631894

[53] Sheu HJ, Wei YC. Effective options trading strategies based on volatility forecasting recruiting investor sentiment. Expert Syst Appl. 2011;38:585–596. doi: 10.1016/j.eswa.2010.07.007

[54] Feuerriegel S, Neumann D. Evaluation of News-Based Trading Strategies. Springer International Publishing. 2014;90:65–74.

[55] Khanal AR, Mishra AK. Is the ‘buying winners and selling losers’ trading strategy profitable in the New Economy. Appl Econ Lett. 2014;21:15:1090–1093. doi: 10.1080/13504851.2014.909569

[56] Zhu H, Jiang ZQ, Li SP, Zhou WX. Profitability of simple technical trading rules of Chinese stock exchange indexes. Physica A. 2015;439:75–84. doi: 10.1016/j.physa.2015.07.032

[57] Serban AF. Combining mean reversion and momentum trading strategies in foreign exchange markets. J Bank Financ. 2010;34:2720–2727. doi: 10.1016/j.jbankfin.2010.05.011

[58] Han CW, Hwang SS, Ryu DJ. Market overreaction and investment strategies. Appl Econ. 2015;47:54:5868–5885.

[59] Farmer JD, Joshi S. The price dynamics of common trading strategies. J Econ Behav Organ. 2002;49:149–171. doi: 10.1016/S0167-2681(02)00065-3

[60] Alvarez-Ramirez J, Suarez R, Ibarra-Valdez C. Trading strategies, feedback control and market dynamics. Physica A. 2003;324:220–226. doi: 10.1016/S0378-4371(02)01857-5

[61] Boboc IA, Dinica MC. An Algorithm for Testing the Efficient Market Hypothesis. PLoS One. 2013;8:e78177 doi: 10.1371/journal.pone.0078177 24205148 10.1371/journal.pone.0078177 PMC3812129

[62] Biondo AE, Pluchino A, Rapisarda A, Helbing D. Are Random Trading Strategies More Successful than Technical Ones? PLoS One. 2013;8:e68344 doi: 10.1371/journal.pone.0068344 23874594 10.1371/journal.pone.0068344 PMC3708927

[63] Zhou WX, Mu GH, Chen W, Sornette D. Investment Strategies Used as Spectroscopy of Financial Markets Reveal New Stylized Facts. PLoS One. 2011;6:e24391 doi: 10.1371/journal.pone.0024391 21935403 10.1371/journal.pone.0024391 PMC3173398

[64] Garzarelli F, Cristelli M, Pompa G, Zaccaria A, Pietronero L. Memory effects in stock price dynamics: evidences of technical trading. Sci Rep. 2014;4:4487 doi: 10.1038/srep04487 24671011 10.1038/srep04487 PMC3967202

[65] Park CH. What do we know about the profitability of technical analysis? J Econ Surv. 2007;21:786–826. doi: 10.1111/j.1467-6419.2007.00519.x

[66] Lillo F, Farmer JD. The Long Memory of the Efficient Market. Stud Nonlinear Dyn E. 2004;8:1.

